# Supplementary material for: New Insights into the Regulatory Role of Ferroptosis in Ankylosing Spondylitis via Consensus Clustering of Ferroptosis-Related Genes and Weighted Gene Co-Expression Network Analysis
Source: Genes (Basel). 2022 Jul 31;13(8):1373. doi: 10.3390/genes13081373 (PMC9407156; doi:10.3390/genes13081373)
Supplement: Supplementary file 1 [file genes-13-01373-s001.zip › Supplementary material legends.pdf]

New insights into the regulatory role of ferroptosis in ankylosing spondylitis via consensus clustering of ferroptosis-related genes and weighted gene co-expression network analysis

Tianhua Rong[123], Ningyi Jia[4], Bingxuan Wu[123], Dacheng Sang[1] and Baoge Liu[123*]

[1] Department of Orthopaedic Surgery, Spine Center, Beijing Tiantan Hospital, Capital Medical University, Beijing, China
[2] National Clinical Research Center for Orthopedics, Sports Medicine & Rehabilitation, Beijing, China
[3] Basic and Translational Medicine Center, China National Clinical Research Center for Neurological Diseases, Beijing, China
[4] Department of Obstetrics and Gynecology, Beijing Tiantan Hospital, Capital Medical University, Beijing, China
* Correspondence: baogeliu@hotmail.com

Supplementary Materials:

Supplementary Figure S1. Heatmap of 276 ferroptosis-related genes plotted by age groups. Red indicates high expression, and blue indicates low expression.

Supplementary Figure S2. Box plot shows the distribution of patient age in the two clusters determined by ferroptosis-related genes.

Supplementary Figure S3. External validation of consensus clustering. (A) Consensus score matrix for the 22 AS samples obtained from dataset GSE141646 when k = 2. (B) Empirical consensus cumulative distribution function plots for k = 2 to 6, indicating that k = 2 or 3 is acceptable. Provided the sample size and interpretability, the optimal k value was also 2.

Supplementary Table S1. The detailed information about the compilation of ferroptosis-related genes from the FerrDb database and PubMed articles.

Supplementary Table S2. Results of leukocyte deconvolution for all the AS samples using the CIBERSORT computational approach.

Supplementary Table S3. The detailed information about the 86 ferroptosis-related genes that expressed differently between the two clusters.

Supplementary Table S4. The gene significance and module membership of all genes in the turquoise module in WGCNA, and the selection of candidate hub genes.

Supplementary Table S5. The information about the nodes, edges, and connectivity in the multifactor regulatory network.
