# Supplementary figures and images for: New Insights into the Regulatory Role of Ferroptosis in Ankylosing Spondylitis via Consensus Clustering of Ferroptosis-Related Genes and Weighted Gene Co-Expression Network Analysis
Source: Genes (Basel). 2022 Jul 31;13(8):1373. doi: 10.3390/genes13081373 (PMC9407156; doi:10.3390/genes13081373)

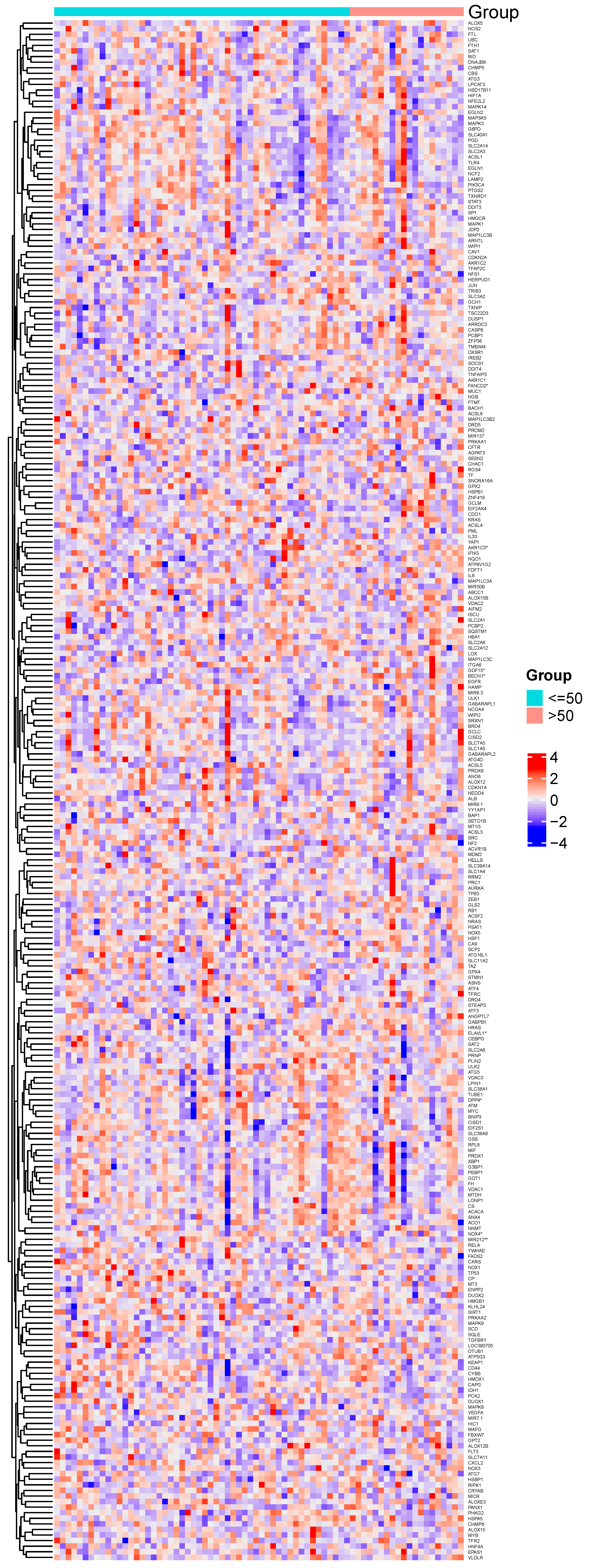

Supplement: Supplementary file 1 [file genes-13-01373-s001.zip › Supplementary Figure S1.jpg]

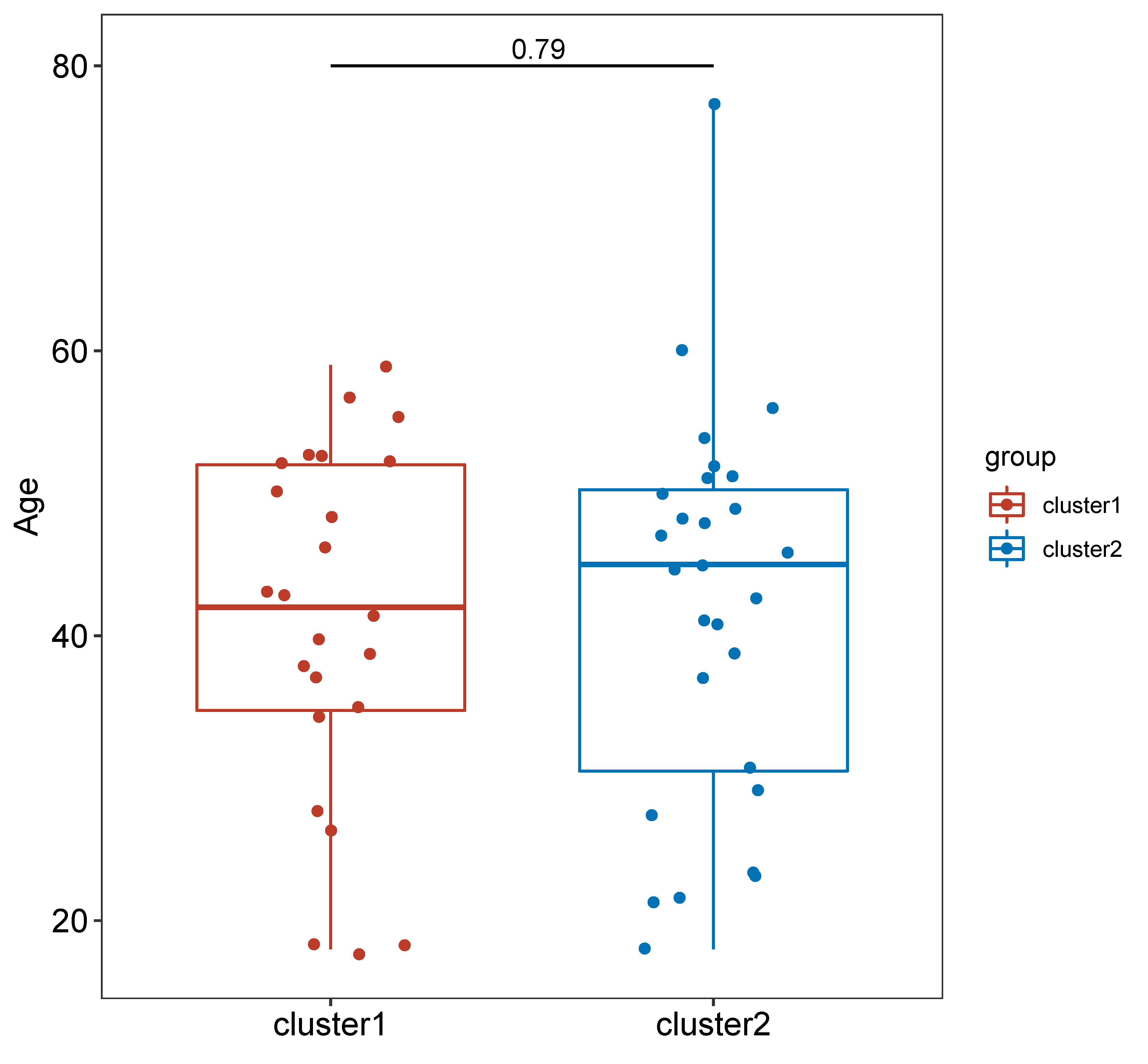

Supplement: Supplementary file 1 [file genes-13-01373-s001.zip › Supplementary Figure S2.jpg]

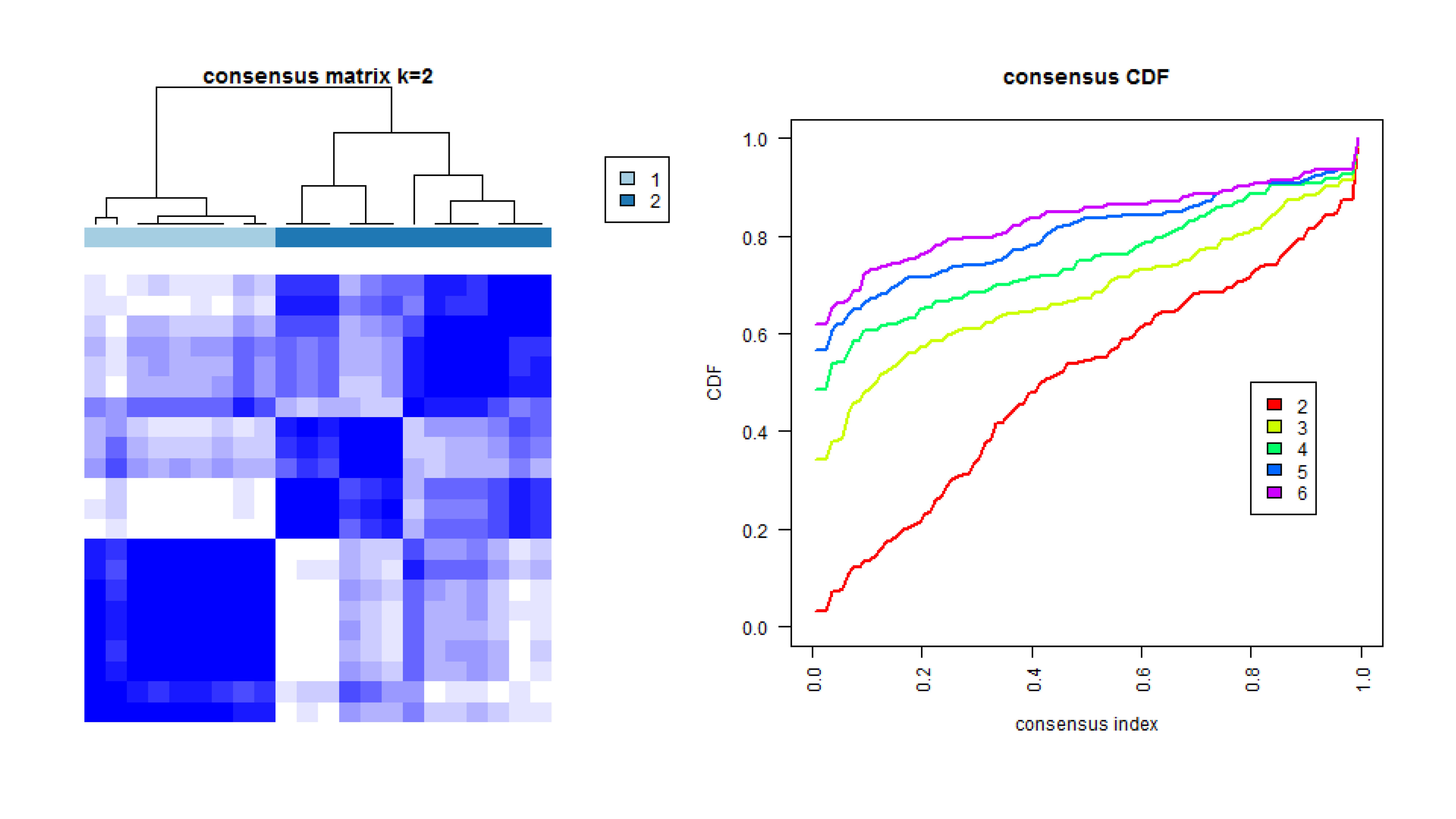

Supplement: Supplementary file 1 [file genes-13-01373-s001.zip › Supplementary Figure S3.jpg]
